# Supplementary figures and images for: Long range segmentation of prokaryotic genomes by gene age and functionality
Source: bioRxiv. 2024 Apr 26:2024.04.26.591304. Preprint. [Version 1] doi: 10.1101/2024.04.26.591304 (PMC11188115; doi:10.1101/2024.04.26.591304)

least common  
genes in  
Sulfolobus

most common  
genes in  
Sulfolobus

←-----→

←-→

$Q^L_{Sulfolobus}$

$Q^H_{Sulfolobus}$

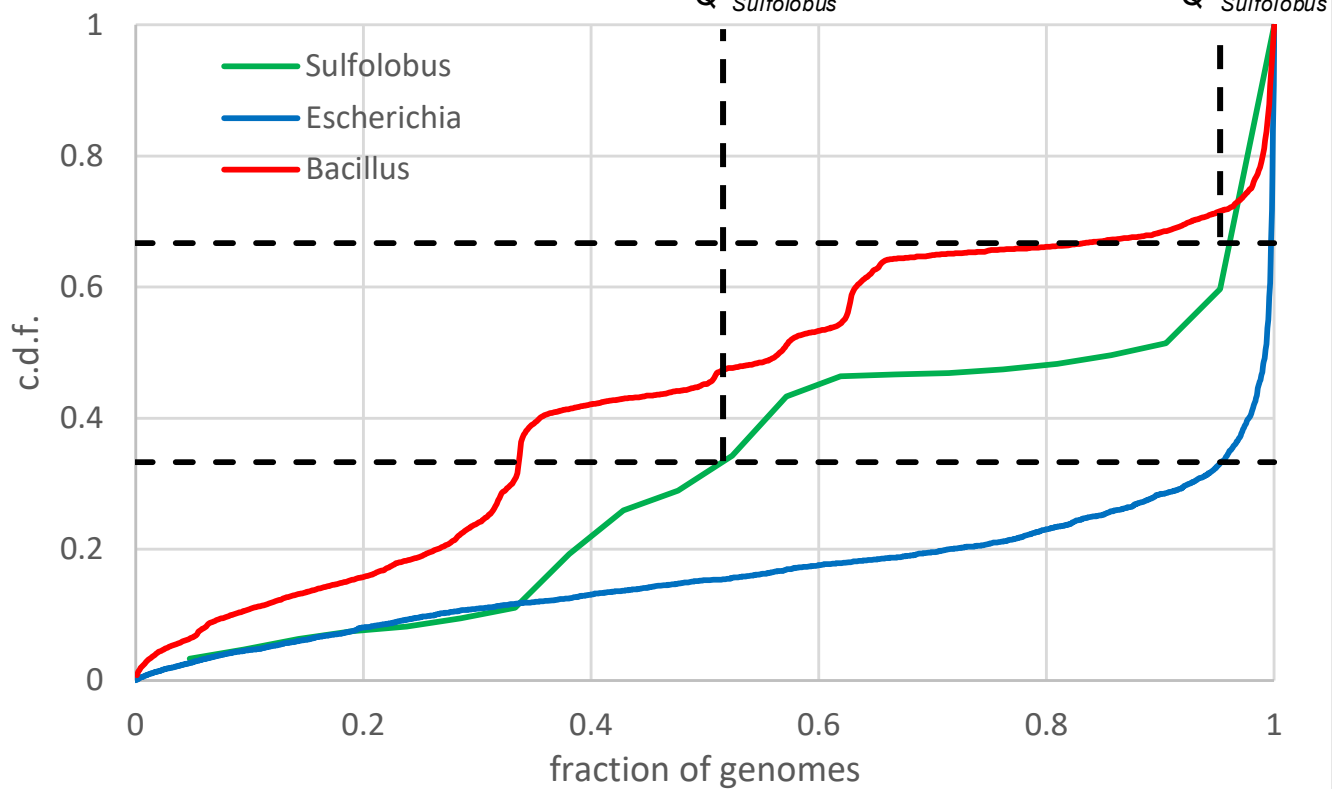

Supplement: Supplement 1 — Cumulative distribution function of the protein cluster commonality (fraction of genomes within a genus where members of the cluster are present) is plotted for aggregated genomes of Sulfolobus (green line), Escherichia (blue line) and Bacillus (red line) genera. Horizontal dotted lines indicate the 1/3rd and 2/3rd quantiles, separating the gene complement into three frequency partitions (the least common genes, intermediate commonality genes, and the most common genes). The intersections of these quantile lines with the cumulative distribution curve for a genus G correspond to the two quantile thresholds, QGL and QGH, allowing the classification of protein clusters (and the respective genes) into the least common (“young”) and the most common (“ancient”) categories (displayed for Sulfolobus). [file media-1.pdf]

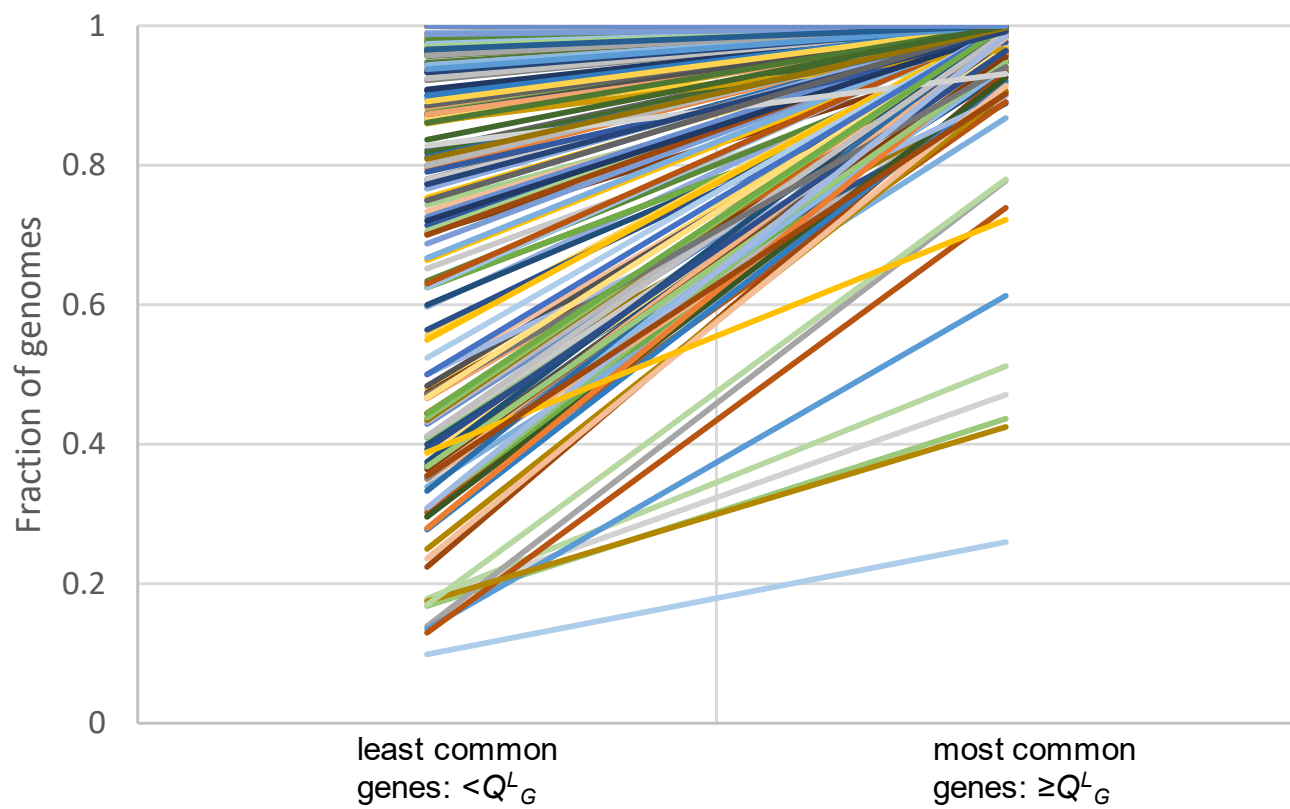

Supplement: Supplement 8 — The vertical axis shows the cluster commonality range (0 to 1) with lines connecting the lower and the upper thresholds for each genus (QGL and QGH, respectively), separating the “young”, intermediate, and “ancient” genes. [file media-8.pdf]

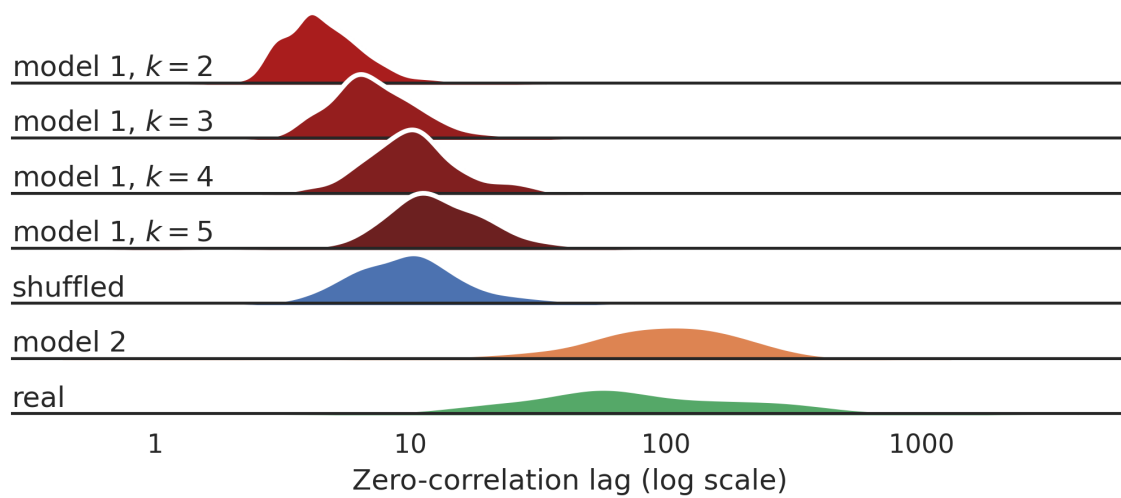

Supplement: Supplement 9 — The distributions (kernel density estimation) of logarithms of zero-correlation lags. Model 1 is the “protected essential genes” model. Model 2 is the “differential mobility and attractors” model. Shuffled genomes are obtained from the real ones by random shuffling of contiguous blocks while keeping their number and sizes preserved. [file media-9.pdf]
