# Supplementary material for: Long range segmentation of prokaryotic genomes by gene age and functionality
Source: bioRxiv. 2024 Apr 26:2024.04.26.591304. Preprint. [Version 1] doi: 10.1101/2024.04.26.591304 (PMC11188115; doi:10.1101/2024.04.26.591304)
Supplement: Supplement 3 — Each circular chromosome is displayed as two concentric rings, showing the average density of ancient (outer ring, green) and young (inner ring, red) genes in chromosome segments that are shown by arcs. The tree shows the approximate evolutionary relationships between Sulfolobus isolates (48). [file media-3.pdf]

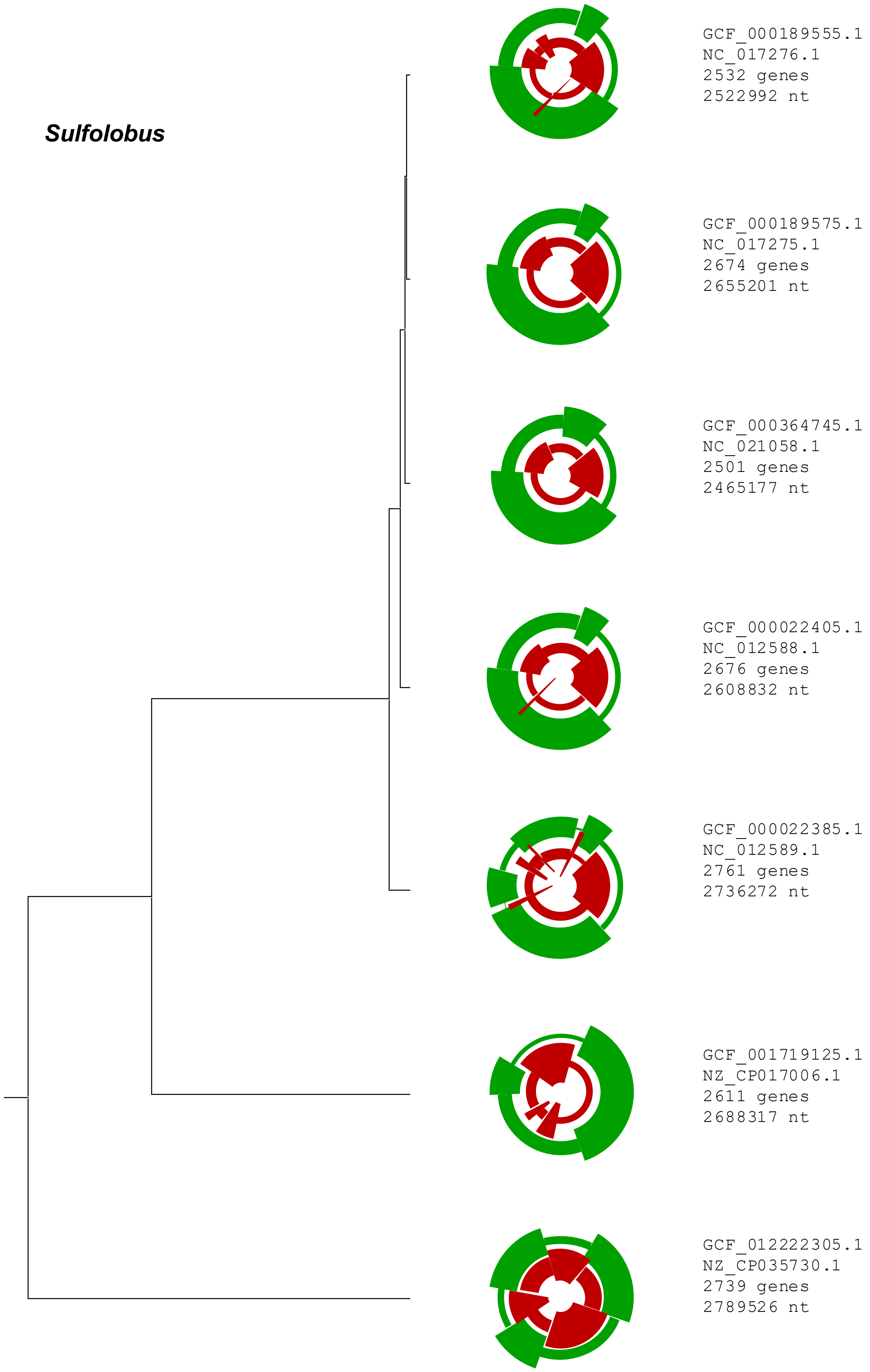
Sulfolobus
GCF_000189555.1
NC_017276.1
2532 genes
2522992 nt
GCF_000189575.1
NC_017275.1
2674 genes
2655201 nt
GCF_000364745.1
NC_021058.1
2501 genes
2465177 nt
GCF_000022405.1
NC_012588.1
2676 genes
2608832 nt
GCF_000022385.1
NC_012589.1
2761 genes
2736272 nt
GCF_001719125.1
NZ_CP017006.1
2611 genes
2688317 nt
GCF_012222305.1
NZ_CP035730.1
2739 genes
2789526 nt
