# Supplementary material for: Long range segmentation of prokaryotic genomes by gene age and functionality
Source: bioRxiv. 2024 Apr 26:2024.04.26.591304. Preprint. [Version 1] doi: 10.1101/2024.04.26.591304 (PMC11188115; doi:10.1101/2024.04.26.591304)
Supplement: Supplement 4 — Each circular chromosome is displayed as two concentric rings, showing the average density of ancient (outer ring, green) and young (inner ring, red) genes in chromosome segments that are shown by arcs. The tree shows the approximate evolutionary relationships between Lactococcus isolates (48). [file media-4.pdf]

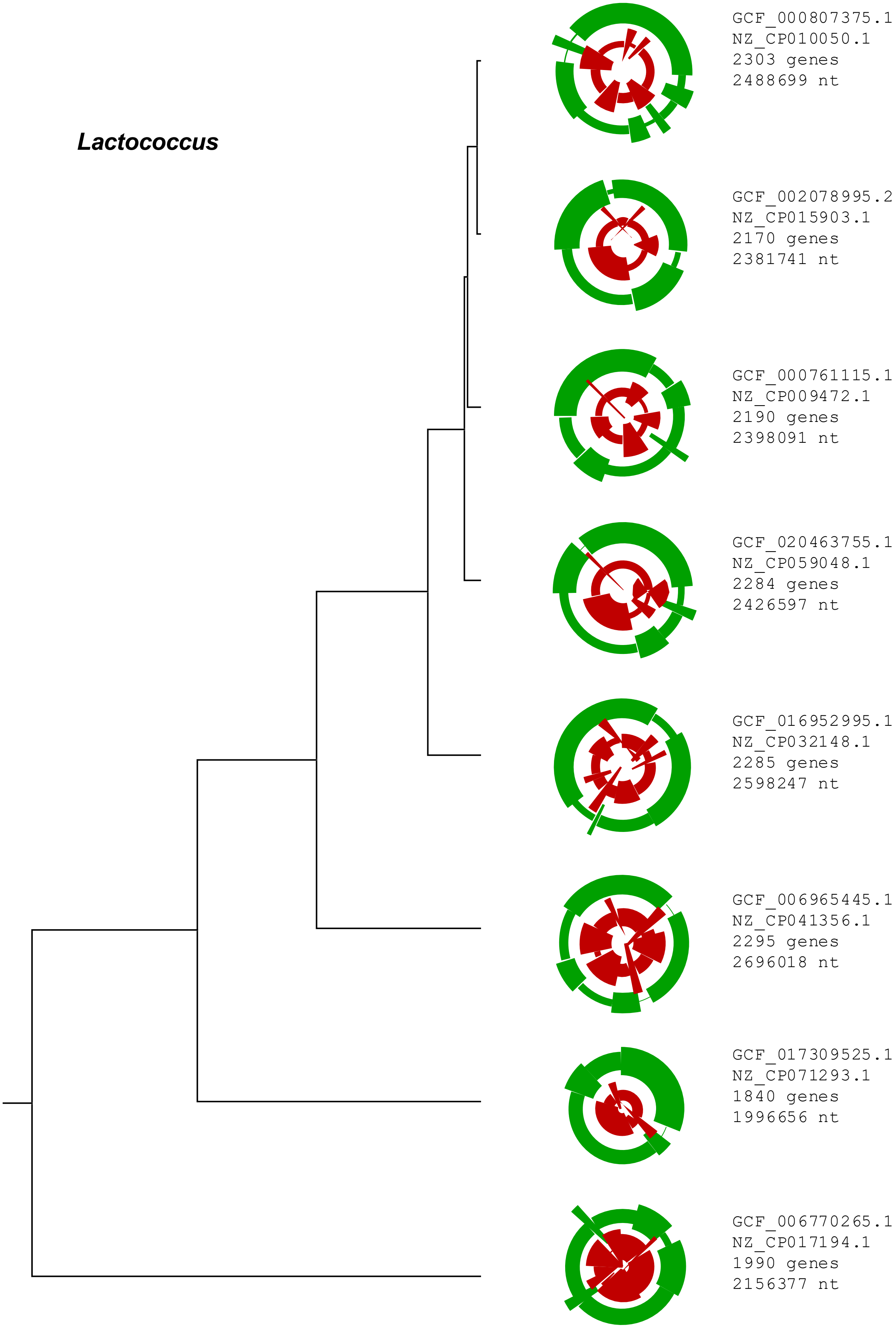

Lactococcus
GCF_000807375.1
NZ_CP010050.1
2303 genes
2488699 nt
GCF_002078995.2
NZ_CP015903.1
2170 genes
2381741 nt
GCF_000761115.1
NZ_CP009472.1
2190 genes
2398091 nt
GCF_020463755.1
NZ_CP059048.1
2284 genes
2426597 nt
GCF_016952995.1
NZ_CP032148.1
2285 genes
2598247 nt
GCF_006965445.1
NZ_CP041356.1
2295 genes
2696018 nt
GCF_017309525.1
NZ_CP071293.1
1840 genes
1996656 nt
GCF_006770265.1
NZ_CP017194.1
1990 genes
2156377 nt
