# Supplementary material for: Long range segmentation of prokaryotic genomes by gene age and functionality
Source: bioRxiv. 2024 Apr 26:2024.04.26.591304. Preprint. [Version 1] doi: 10.1101/2024.04.26.591304 (PMC11188115; doi:10.1101/2024.04.26.591304)

Sulfolobus_islandicus_REY15A

Sulfolobus_islandicus_HVE104

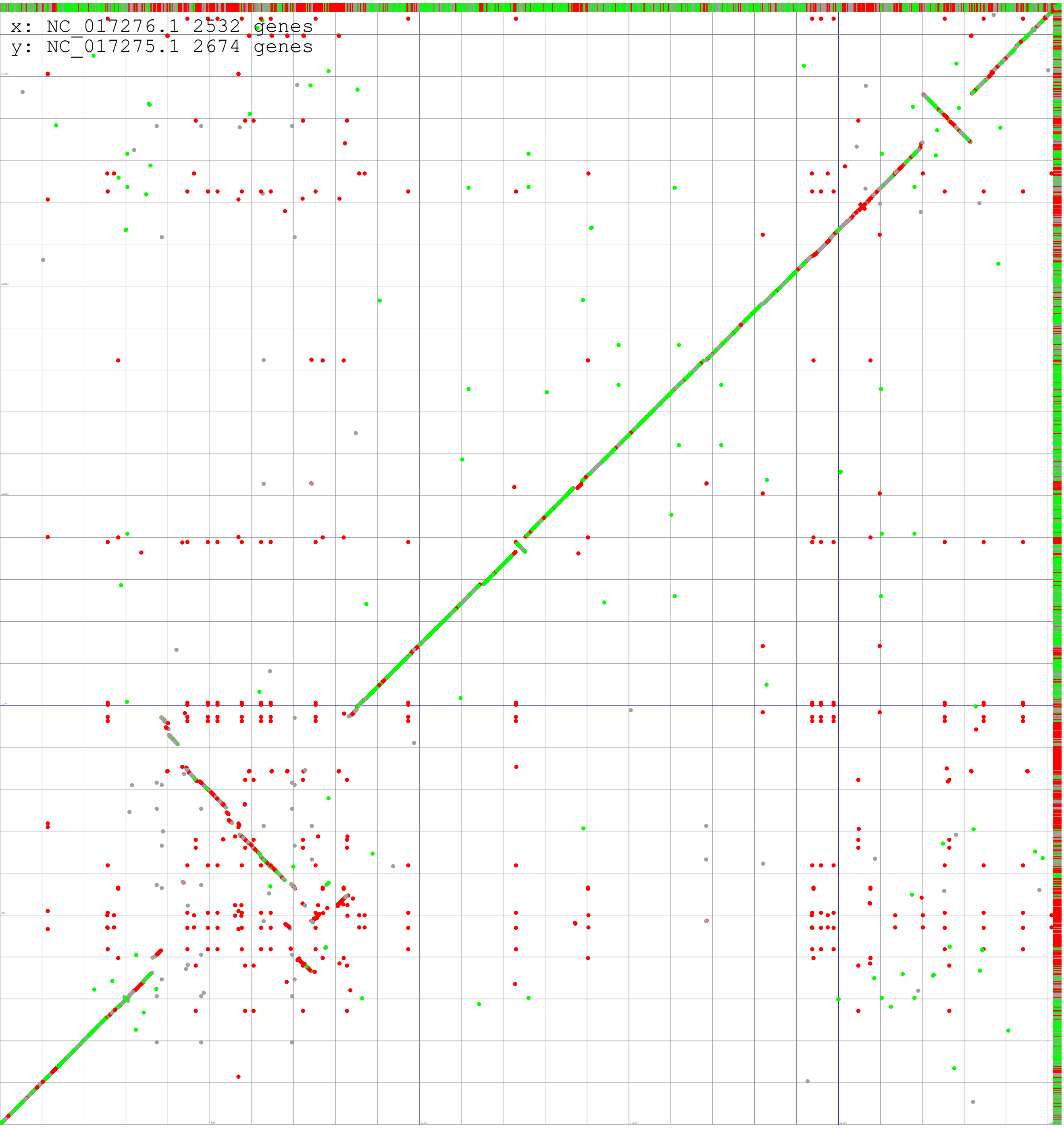

Sulfolobus_islandicus_REY15A

Sulfolobus_islandicus_LAL141

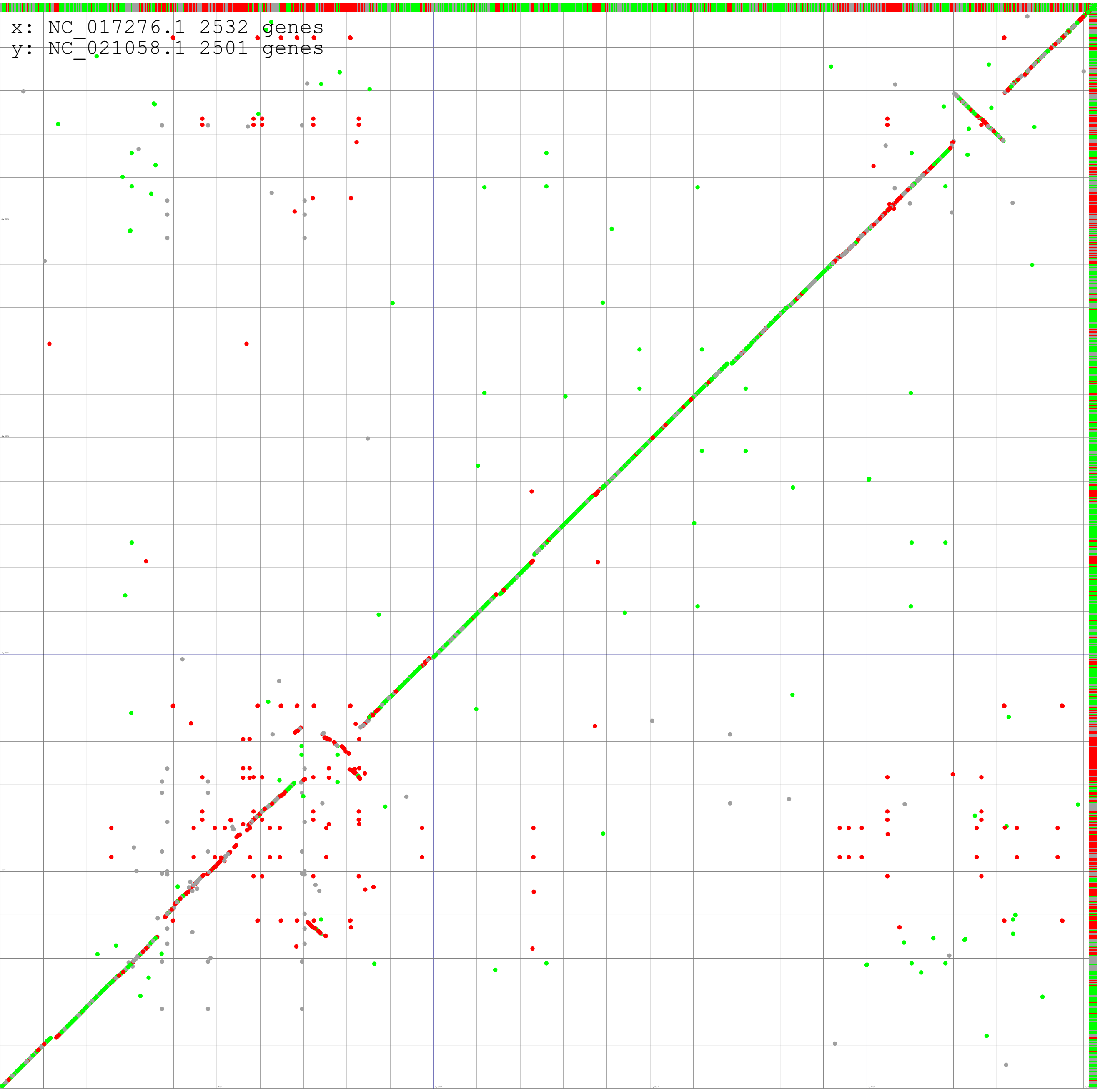

Sulfolobus_islandicus_REY15A

Sulfolobus_islandicus_M.14.25

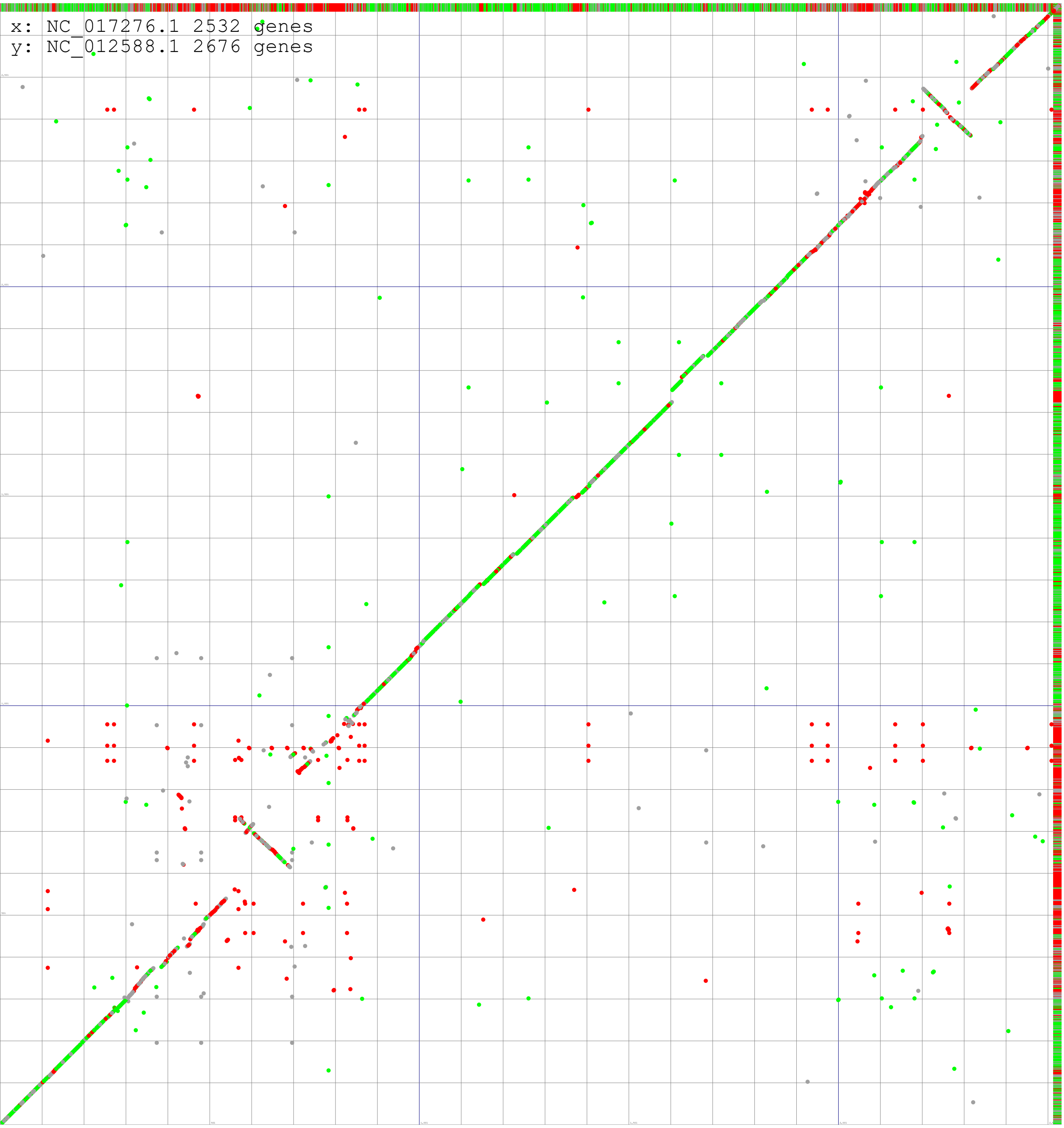

Sulfolobus_islandicus_REY15A

Sulfolobus_islandicus_L.S.2.15

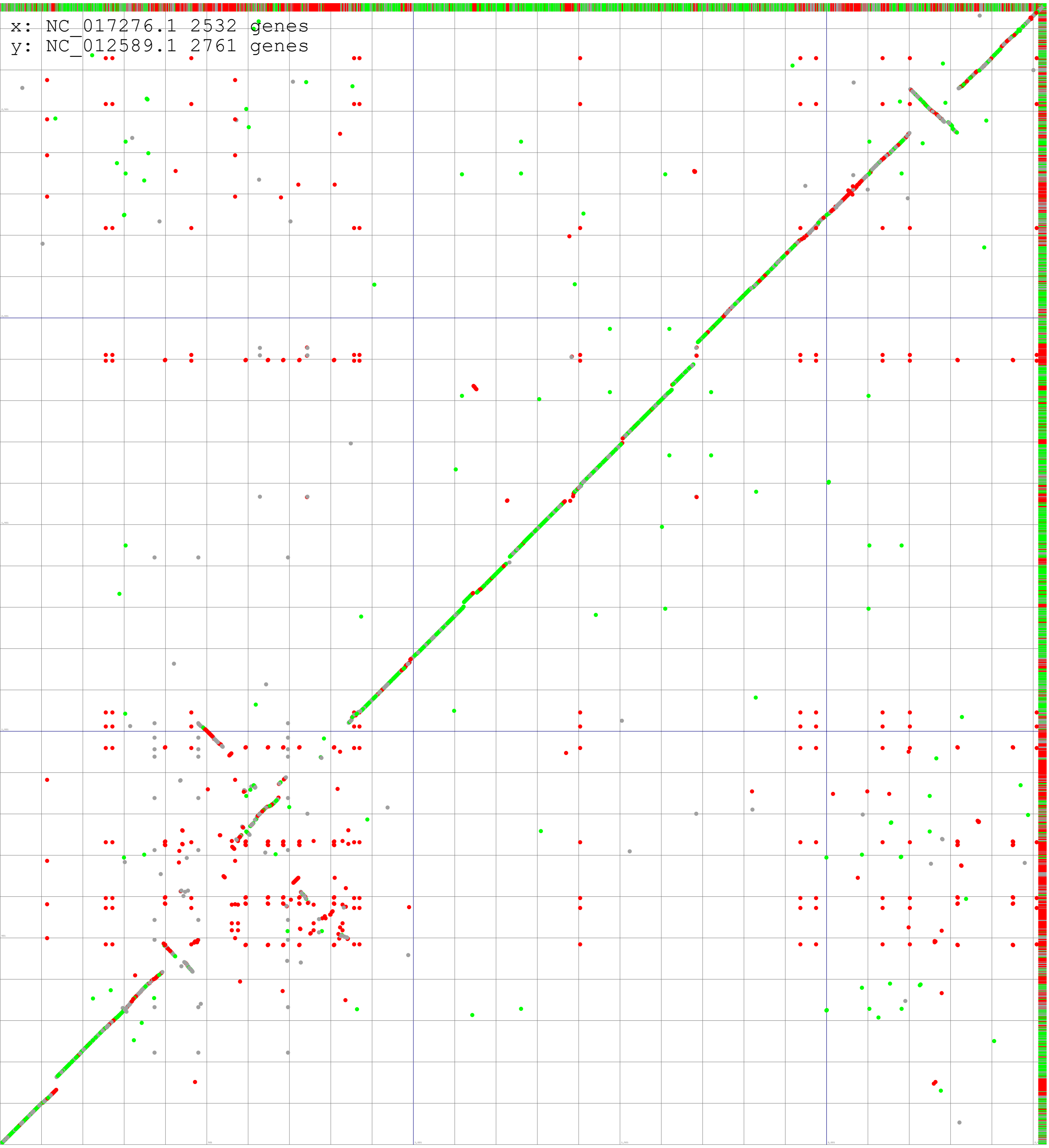

Sulfolobus_islandicus_REY15A

Sulfolobus_A20

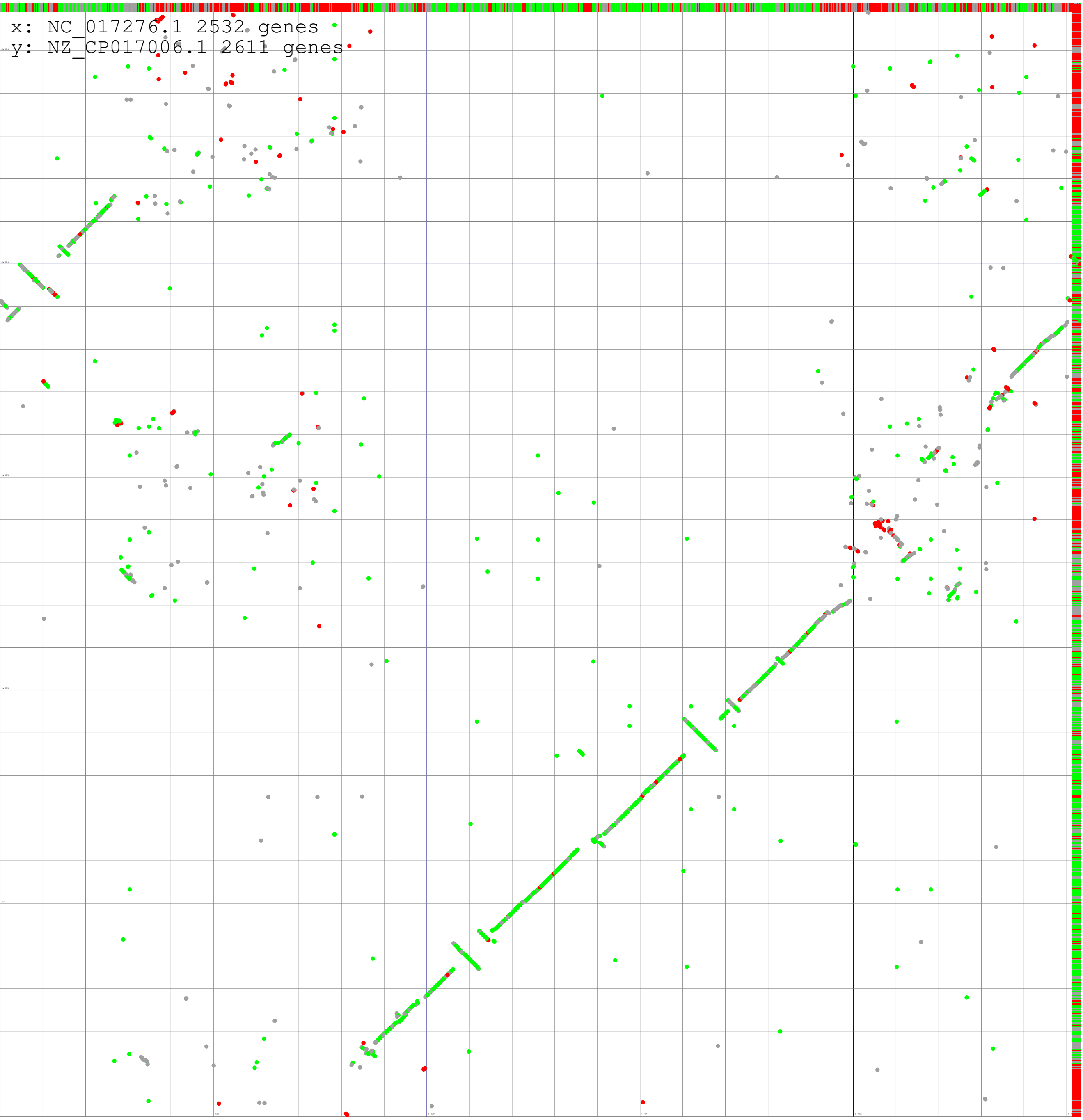

Sulfolobus_islandicus_REY15A

Sulfolobus_S-194

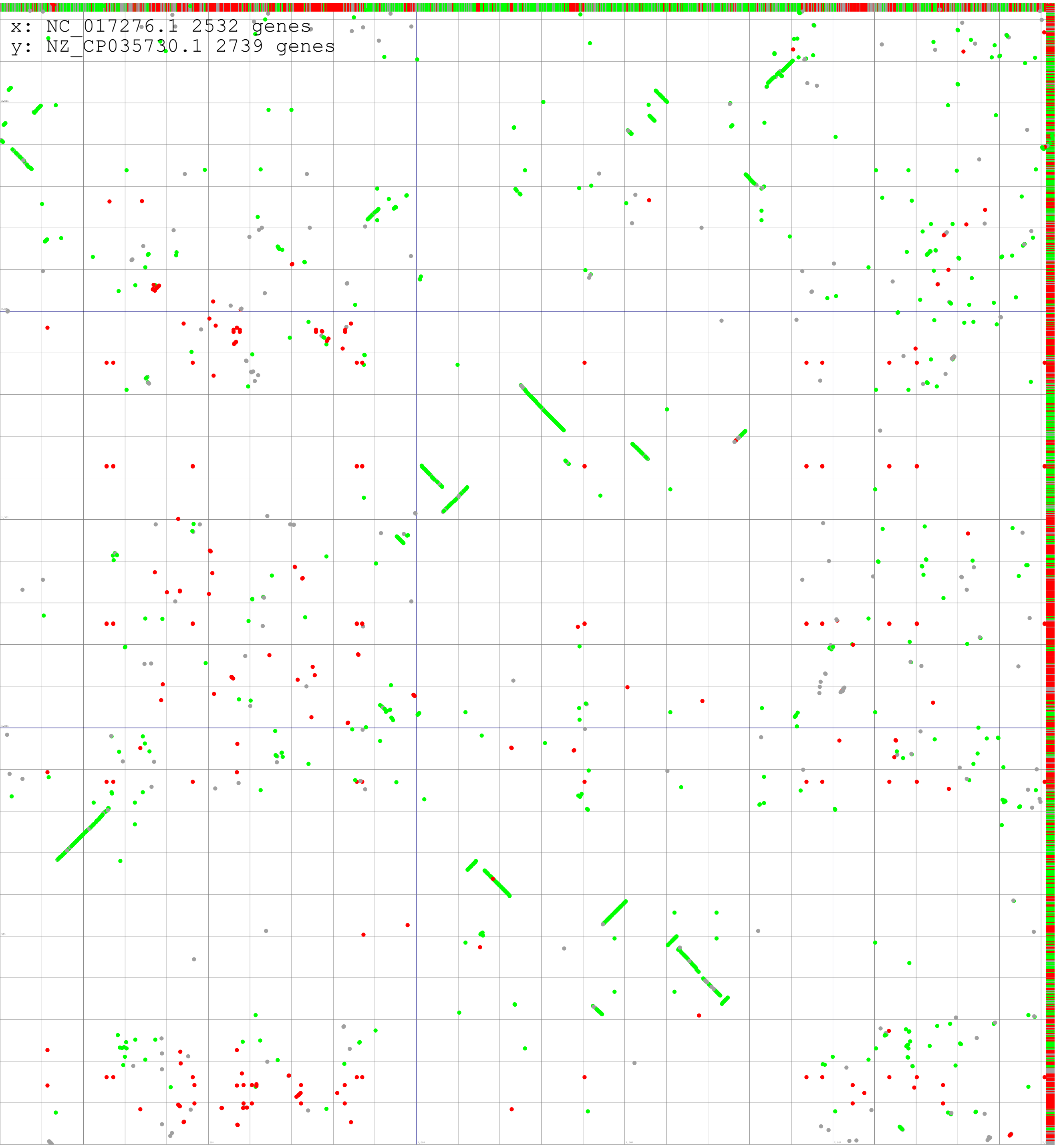

Supplement: Supplement 5 — Dots indicate the locations of shared genes (belonging to the same 0.5 amino acid similarity clusters) between two genomes. The color of the dots and the corresponding bars on the horizontal and the vertical edges of the rectangle indicate the estimated evolutionary age of the gene: green, ancient; gray, intermediate; red, young. Panels are arranged in the order of increasing evolutionary distance from S. islandicus REY15A (on the horizontal axis in all panels). [file media-5.pdf]
