# Supplementary material for: Long range segmentation of prokaryotic genomes by gene age and functionality
Source: bioRxiv. 2024 Apr 26:2024.04.26.591304. Preprint. [Version 1] doi: 10.1101/2024.04.26.591304 (PMC11188115; doi:10.1101/2024.04.26.591304)

Lactococcus_lactis_sub_lactis_S0

Lactococcus_lactis_sub_lactis_UC08

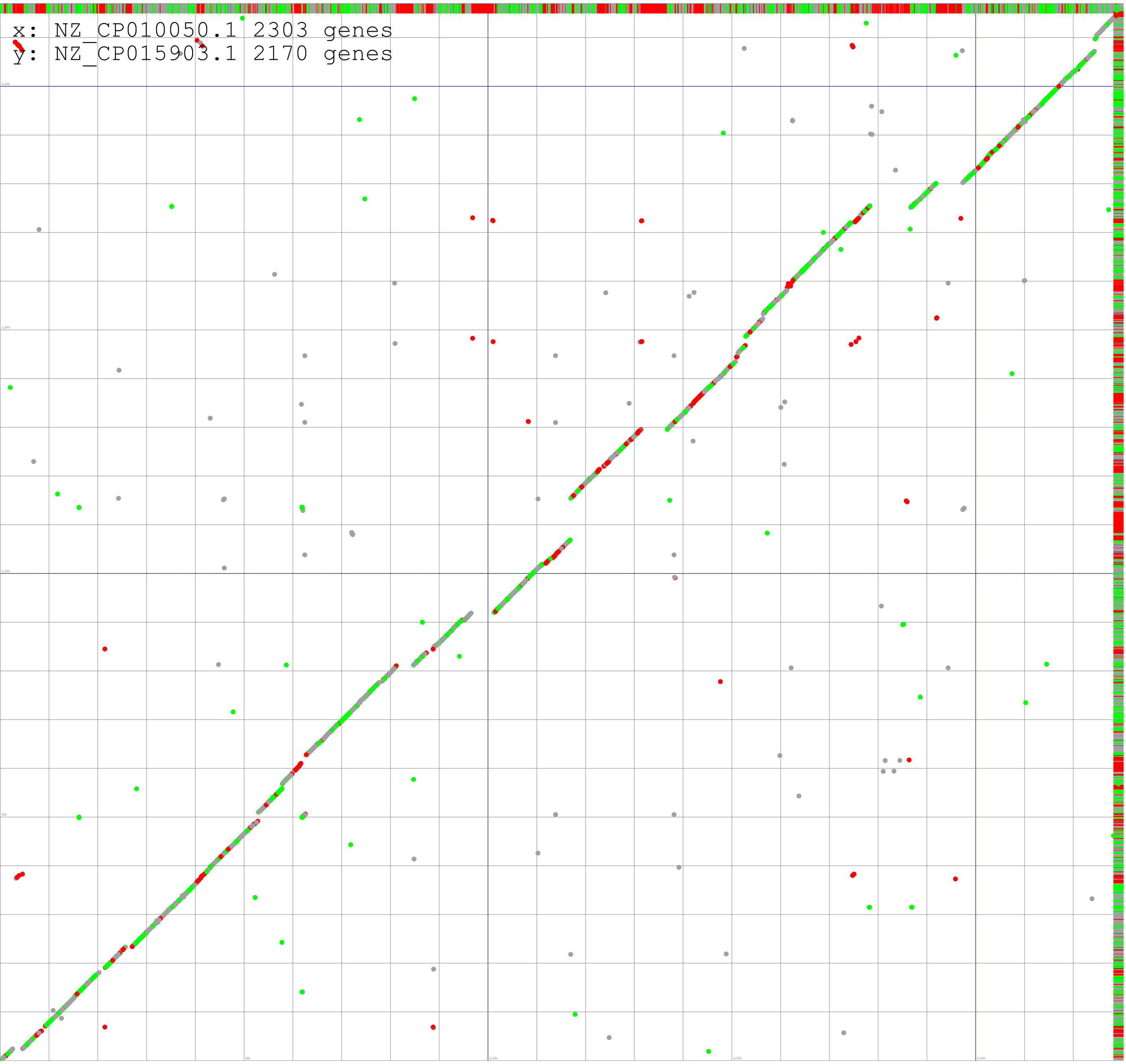

Lactococcus_lactis_sub_lactis_S0

Lactococcus_lactis_AI06

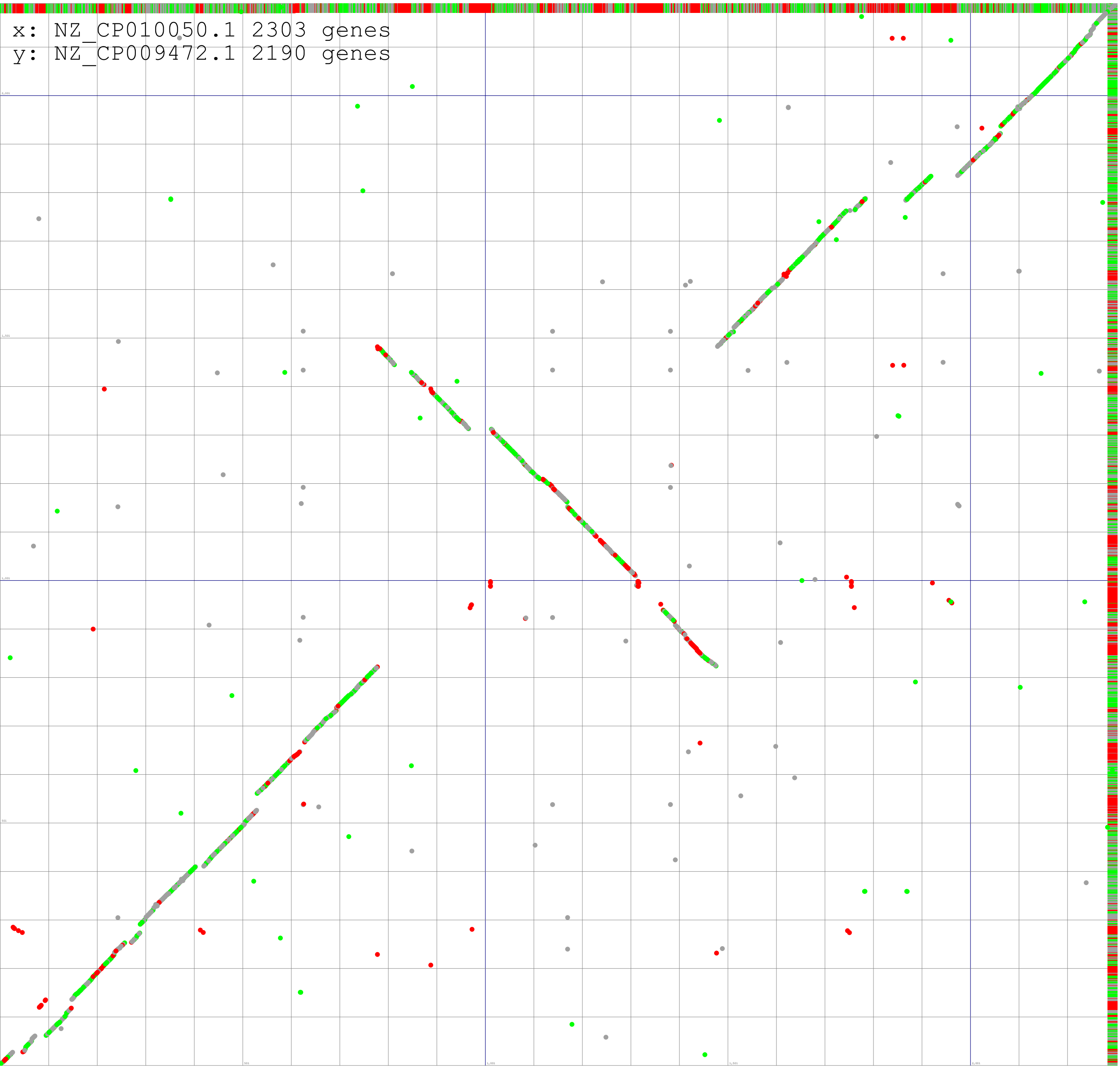

Lactococcus_lactis_sub_lactis_S0

Lactococcus_lactis_LAC460

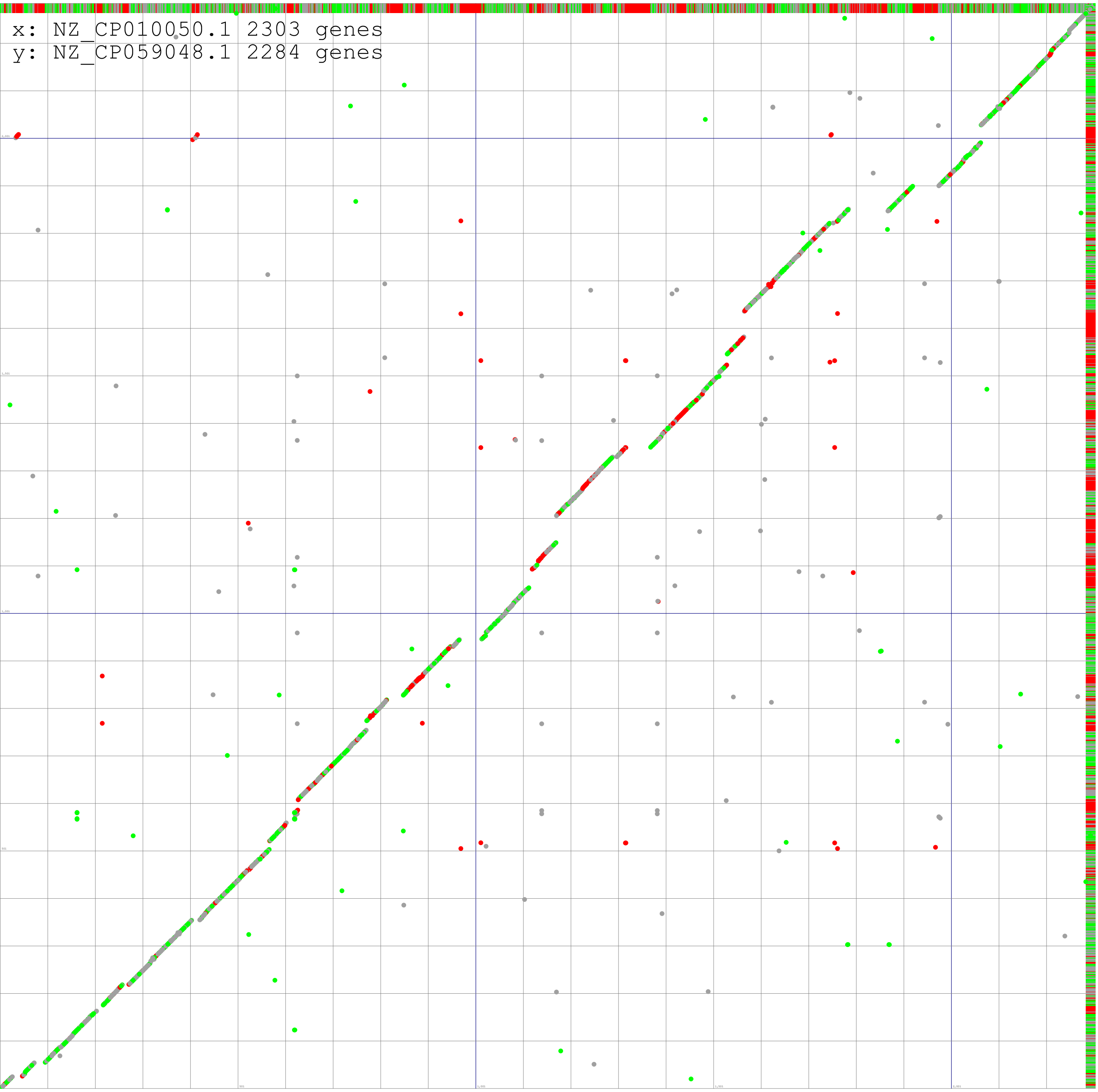

Lactococcus_lactis_sub_lactis_S0

Lactococcus_cremoris_1196

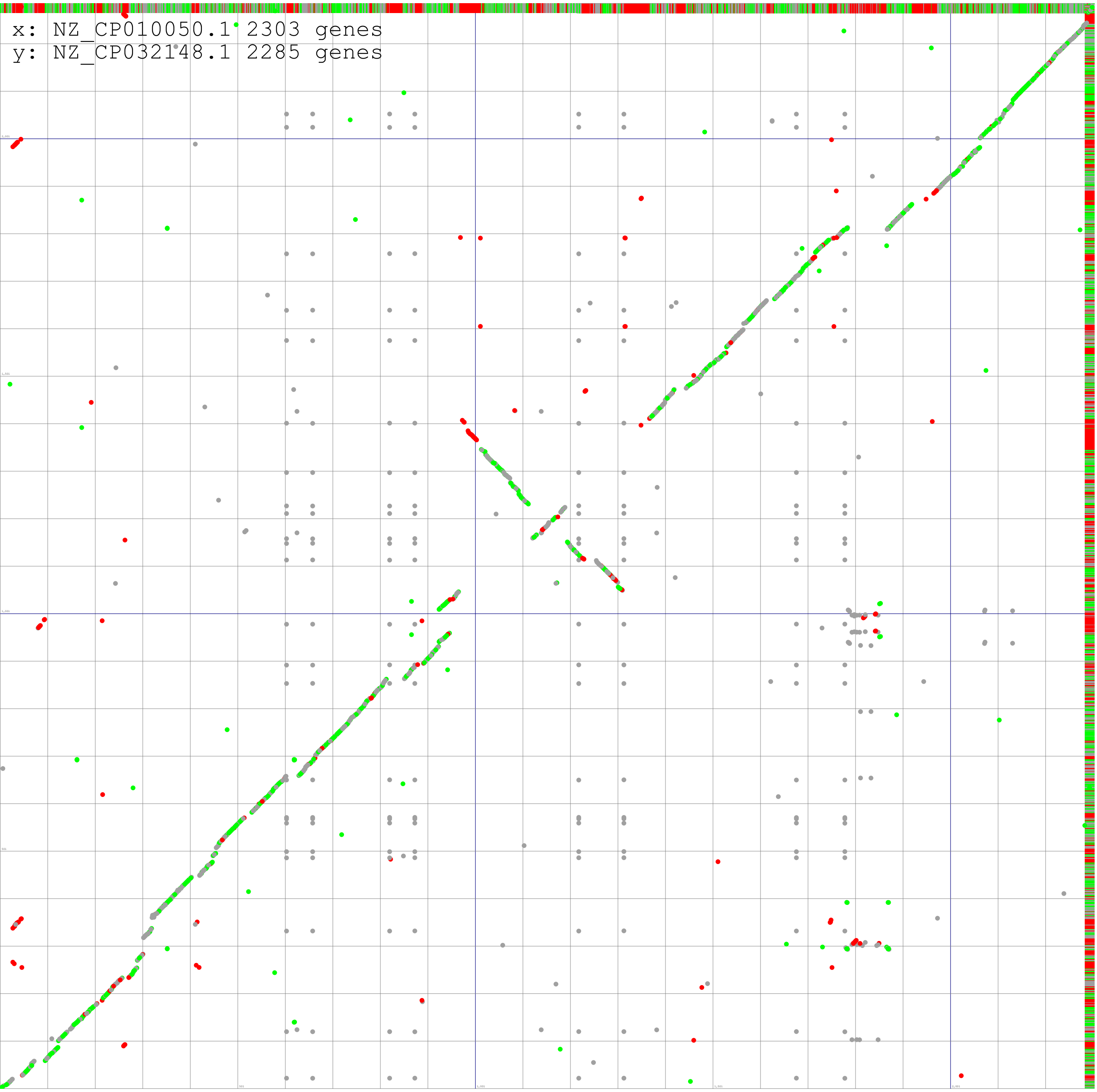

Lactococcus_lactis_sub_lactis_S0

Lactococcus_KACC_19320

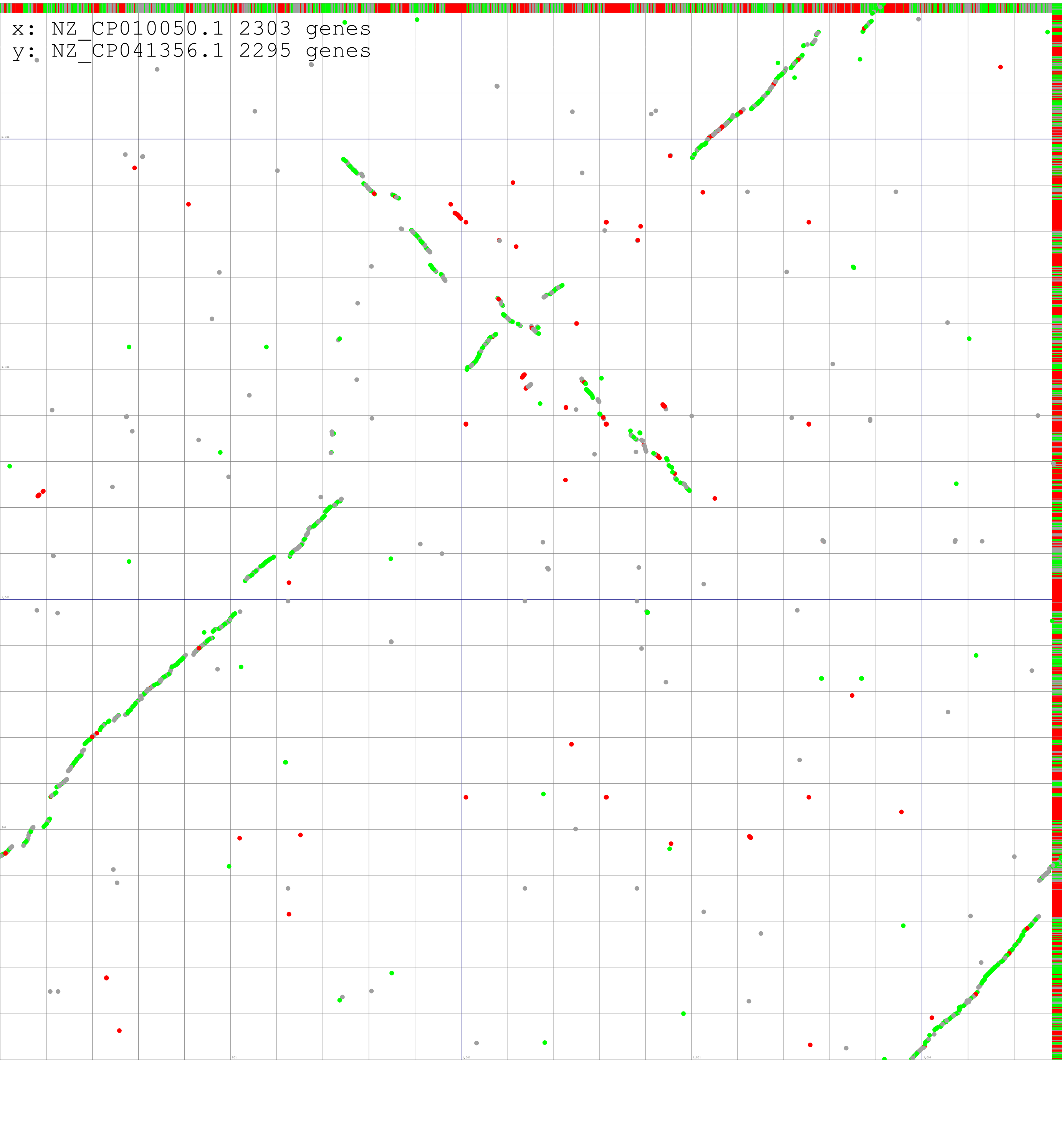

Lactococcus_lactis_sub_lactis_S0

Lactococcus_LG1074

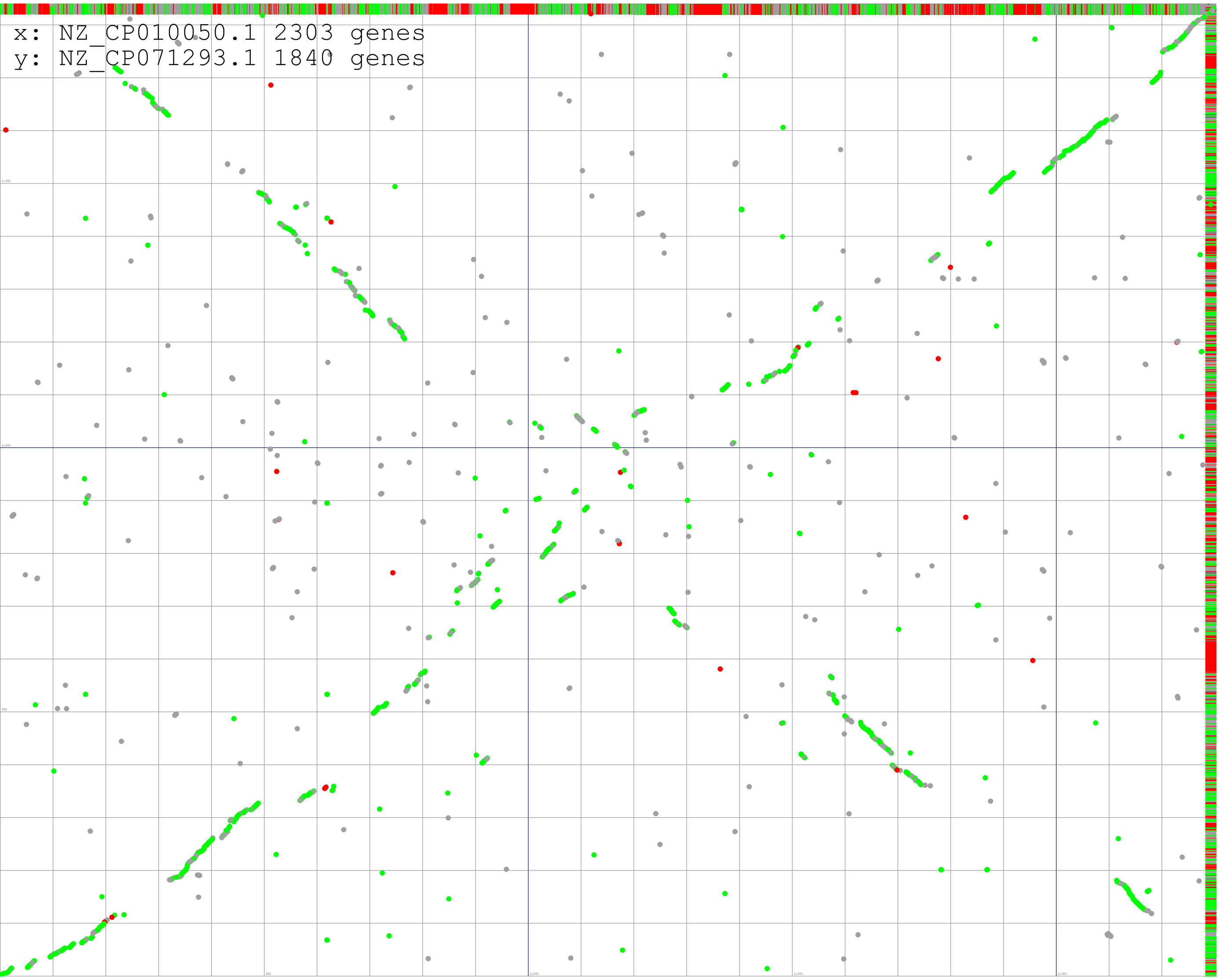

Lactococcus_lactis_sub_lactis_S0

Lactococcus_carnosus_TMW_21612

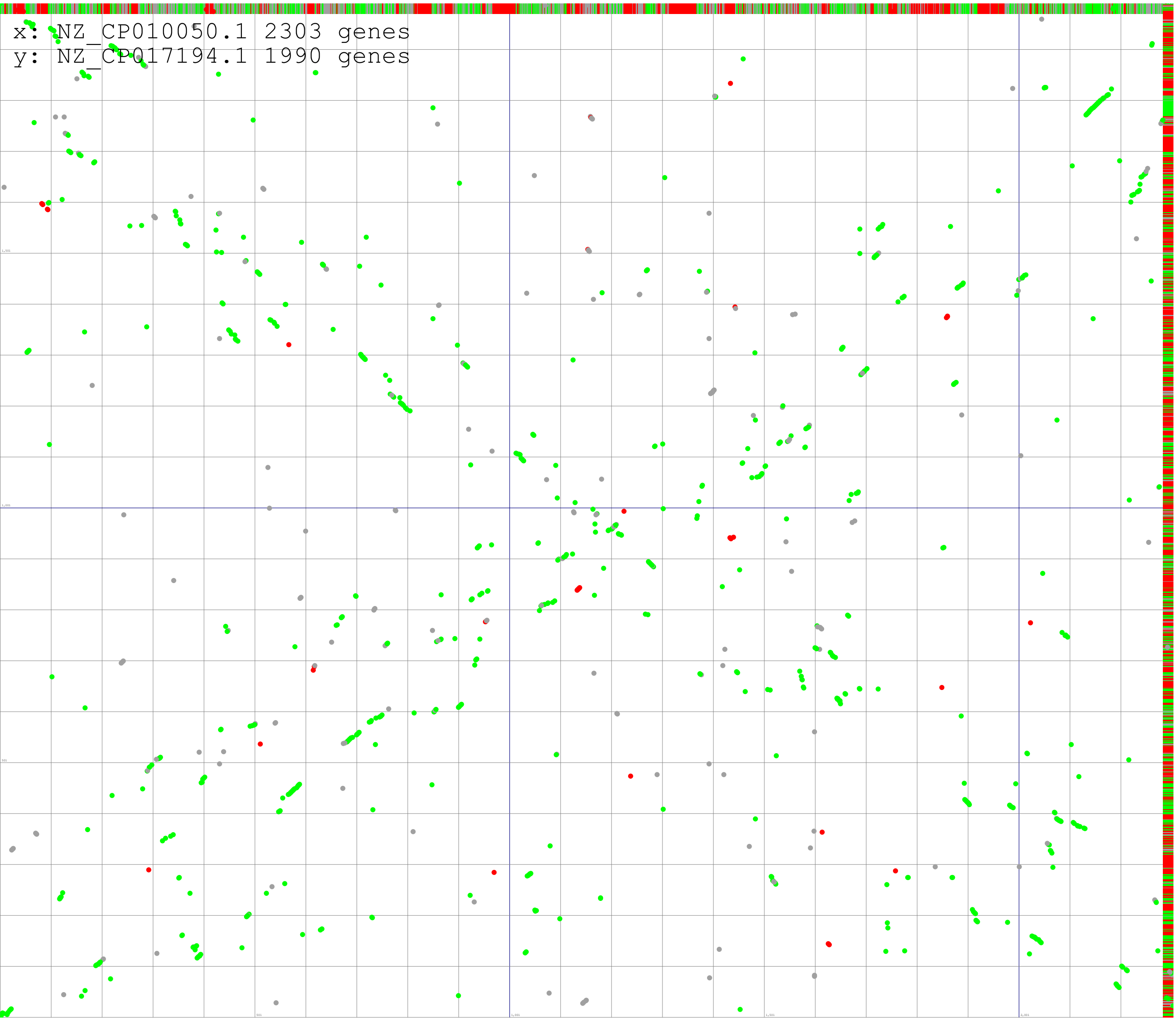

Supplement: Supplement 6 — Dots indicate the locations of shared genes (belonging to the same 0.5 amino acid similarity clusters) between two genomes. The color of the dots and the corresponding bars on the horizontal and the vertical edges of the rectangle indicate the estimated evolutionary age of the gene: green, ancient; gray, intermediate; red, young. Panels are arranged in the order of increasing evolutionary distance from L. lactis subsp. lactis S0 (on the horizontal axis in all panels). [file media-6.pdf]
