# Supplementary material for: Long range segmentation of prokaryotic genomes by gene age and functionality
Source: bioRxiv. 2024 Apr 26:2024.04.26.591304. Preprint. [Version 1] doi: 10.1101/2024.04.26.591304 (PMC11188115; doi:10.1101/2024.04.26.591304)
Supplement: Supplement 7 — Comparison of the relative enrichment of enriched segments; the number of segments, average length of enriched segments, fraction of genome in enriched segments, the average density of tagged genes in the enriched segments and the fraction of tagged genes across all enriched segments in simulated and observed chromosomes (y- and x-axis, respectively). [file media-7.pdf]

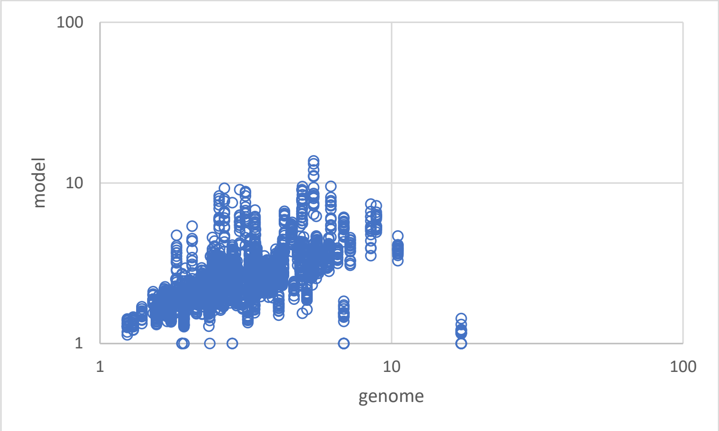

enrichment

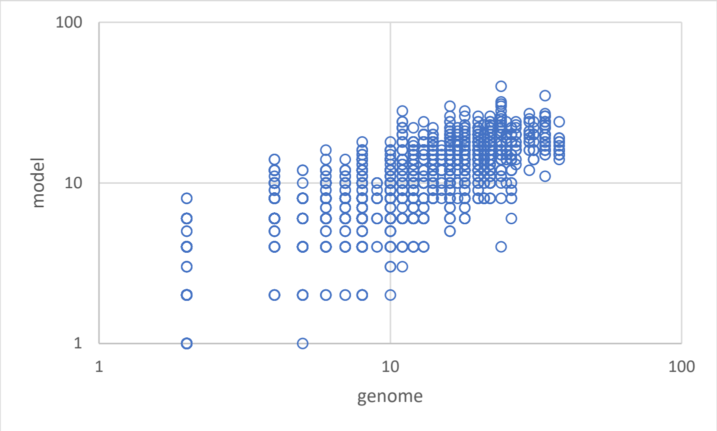

no. of segments

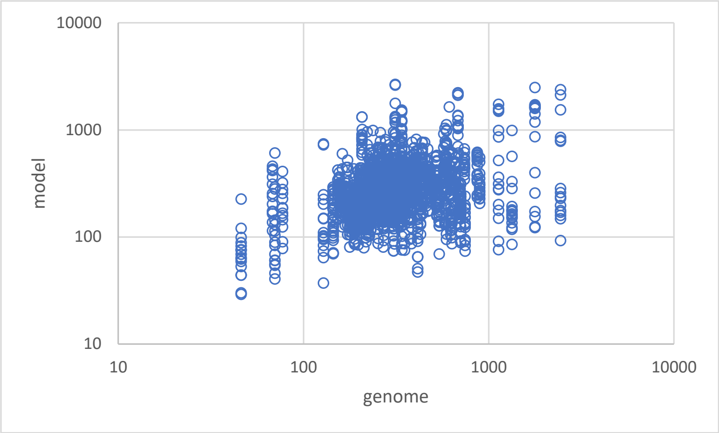

enriched segment length

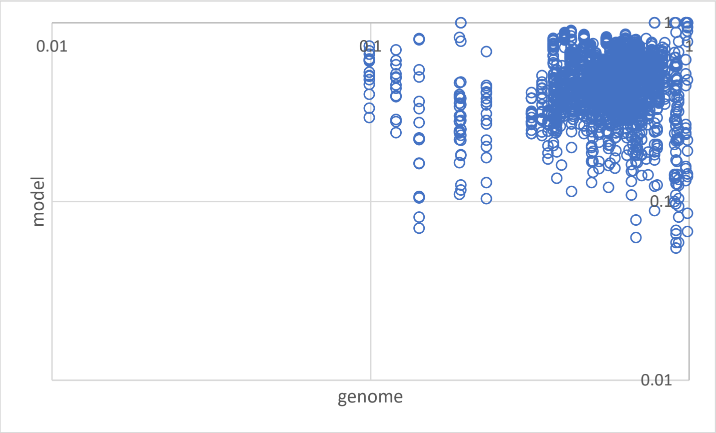

fraction of genome in enriched segments

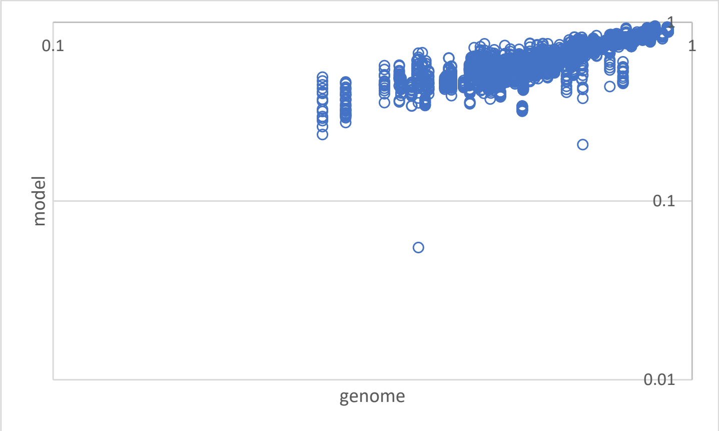

density of enriched segments

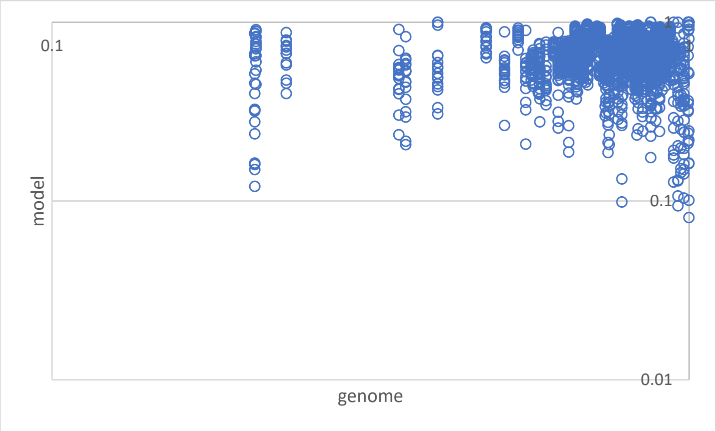

fraction of ancient genes in enriched segments
