# Supplementary material for: Long range segmentation of prokaryotic genomes by gene age and functionality
Source: bioRxiv. 2024 Apr 26:2024.04.26.591304. Preprint. [Version 1] doi: 10.1101/2024.04.26.591304 (PMC11188115; doi:10.1101/2024.04.26.591304)
Supplement: Supplement 16 [file NIHPP2024.04.26.591304v1-supplement-16.pdf]

S7 Table. Simulation of gene order evolution under the differential mobility of genes and differential attraction of intergenic spacers: segmentation of simulated chromosomes

Quantitative characterization of the simulated chromosome partitioning into segments, enriched and depleted with low mobility genes. Data is shown for a randomly sampled the state in multiple independent model runs. Quantitative characteristics of ancient gene segmentation in natural chromosomes are shown for comparison.
